# Highly Flexible and Efficient Fabric-Based Organic Light-Emitting Devices for Clothing-Shaped Wearable Displays

**DOI:** 10.1038/s41598-017-06733-8

**Published:** 2017-07-25

**Authors:** Seungyeop Choi, Seonil Kwon, Hyuncheol Kim, Woohyun Kim, Jung Hyun Kwon, Myung Sub Lim, Ho Seung Lee, Kyung Cheol Choi

**Affiliations:** Korea Advanced Institute of Science and Technology (KAIST), School of Electrical Engineering, Daejeon, 34141 Republic of Korea

## Abstract

Recently, the role of clothing has evolved from merely body protection, maintaining the body temperature, and fashion, to advanced functions such as various types of information delivery, communication, and even augmented reality. With a wireless internet connection, the integration of circuits and sensors, and a portable power supply, clothes become a novel electronic device. Currently, the information display is the most intuitive interface using visualized communication methods and the simultaneous concurrent processing of inputs and outputs between a wearer and functional clothes. The important aspect in this case is to maintain the characteristic softness of the fabrics even when electronic devices are added to the flexible clothes. Silicone-based light-emitting diode (LED) jackets, shirts, and stage costumes have started to appear, but the intrinsic stiffness of inorganic semiconductors causes wearers to feel discomfort; thus, it is difficult to use such devices for everyday purposes. To address this problem, a method of fabricating a thin and flexible emitting fabric utilizing organic light-emitting diodes (OLEDs) was developed in this work. Its flexibility was evaluated, and an analysis of its mechanical bending characteristics and tests of its long-term reliability were carried out.

## Introduction

A paradigm shift in the shape and form of electronic items has moved from rigid and hard electronics, such as personal computers and smart phones, to flexible electronics, including wearable devices. This phenomenon is a natural course driven by the desire of users who want more comfortable and convenient devices^1, 2^. Accordingly, wearable electronics based on flexible technologies have been actively researched and developed, with enormous interest^3–5^. Eyeglasses and lenses for augmented reality as well as smart watches are typical examples, and functional clothing is also receiving considerable amounts of attention. If wearable and smart devices are inserted into everyday clothing, these will make our clothes more stylish and functional. In particular, visual information displays are among the key parts of wearable electronics, allowing communication between a wearer and wearable appliances. The sight-dependence of a human, among the five senses of sight, touch, hearing, taste, and smell, is nearly 90%. Therefore, for aesthetic and functional factors as well as intuitive communications with smart electronics, the achievement of clothing-based information displays should take precedence over other wearable devices.

There are three major ways to achieve actual clothing-shaped information displays. First, a display panel can be attached onto a piece of clothing. This is the simplest method, but it has a negative effect on the flexibility of the fabric because conventional plastic substrates for flexible displays are not as pliable as fabrics. The second method is by the fabrication of light-emitting fiber^6^. Fiber-shaped organic light-emitting diodes (OLED) utilizing rotational thermal evaporation and a polyimide (PI) coated silica fiber^7^ represented the first attempt at realizing an emissive fiber, and light-emitting coaxial nanofiber using an ionic transition metal complex^8^ was also reported. To overcome the low emission performance of fiber-based devices, a dip-coating method capable of depositing layers uniformly was used for the polymer light-emitting diode (PLED) fabrication process, and the dip-coated fiber PLED showed luminance of more than 1,400 nit^9^. However, weaving and integration with fiber-based transistors^10–12^ remain as unsolved problems. The last method which can be used is the direct fabrication of an emitting device onto a fabric. This method may have a very useful advantage in that the flexibility of fabric materials is not greatly affected by a display panel, as the substrate is not made of plastic but is an actual fabric. In previous research, an alternating-current powder electroluminescent (EL) device on a polyethylene terephthalate (PET) mesh fabric showed luminance of 44 cd/m^2^ at 440 V and 40 Hz^13^. Fluorescent OLEDs on a soft fabric, reported later, showed greatly improved performance with luminance and current efficiency exceeding 6,000 cd/m^2^ and 8 cd/A, respectively^14^, and a fabric-based OLED with good long-term reliability was also reported^15^. Recently, solution-processed PLEDs were also reported in relation to a simple fabrication process. These low-cost devices could be mass-produced by the roll-to-roll (R2R) process^16^.

Various works have demonstrated the feasibility of light-emitting devices on actual fabrics. However, light-emitting devices remain difficult to be used as wearable displays due to their low luminance and low efficiency. It is also difficult to maintain their performance under harsh mechanical stress conditions because the very high surface roughness of a fabric prevents the operation of nm-thick OLEDs. Therefore, solutions to these two main issues, device performance and surface morphology, are required to achieve clothing-shaped displays. In this paper, the fabrication of a highly efficient and pliable on-fabric OLED is introduced and its characteristics are described. In addition, mechanical analyses of the fabric-based device are conducted and the results discussed.

## Results and Discussion

**Figure 1:**
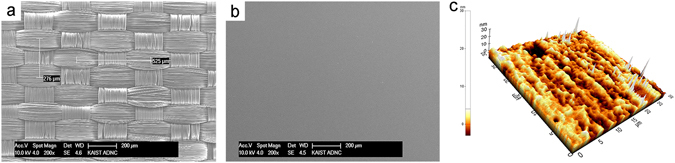
Scanning electron microscope (SEM) and atomic force microscope (AFM) images of the fabric before and after the lamination process: (**a,b**) SEM image of the fabric surface (**a**) before planarization, and (**b**) after planarization. (**c**) Three-dimensional AFM image of the fabric surface after planarization.

The scanning electron microscope (SEM) images in Fig. 1(a) and Supplementary Figure S1 show a woven fabric structure and bundles of fibers. A conventional fabric has a bumpy surface morphology with hundreds of μm-scale bumps. Such a rough surface is not suitable for the fabrication of OLEDs. Therefore, the fabric substrate in this work was woven tightly with fine thread to reduce the surface roughness, and additive planarization of the fabric was conducted to ensure the operation of a 200-nm-thick OLED. The surface roughness after planarization should be a few nm to prevent leakage current and electrical shorting. Thus, thermal lamination utilizing a roll-to-roll process with a thin planarization layer and the fabric was implemented to form a flat surface. As shown in Fig. 1b and c, the surface of the fabric after the planarization step showed an extremely low root-mean-square surface roughness value (R_q_) of 2.073 nm. The mechanical stiffness of the fabric before and after planarization was evaluated by a cantilever test^17^ following the ISO 4064:2011 specification (Supplementary Figure S2). The planarization sheet slightly affected the flexibility of the fabric; however, the planarized fabric was still very pliable as compared to the plastic substrates typically used for flexible OLEDs.

**Figure 2:**
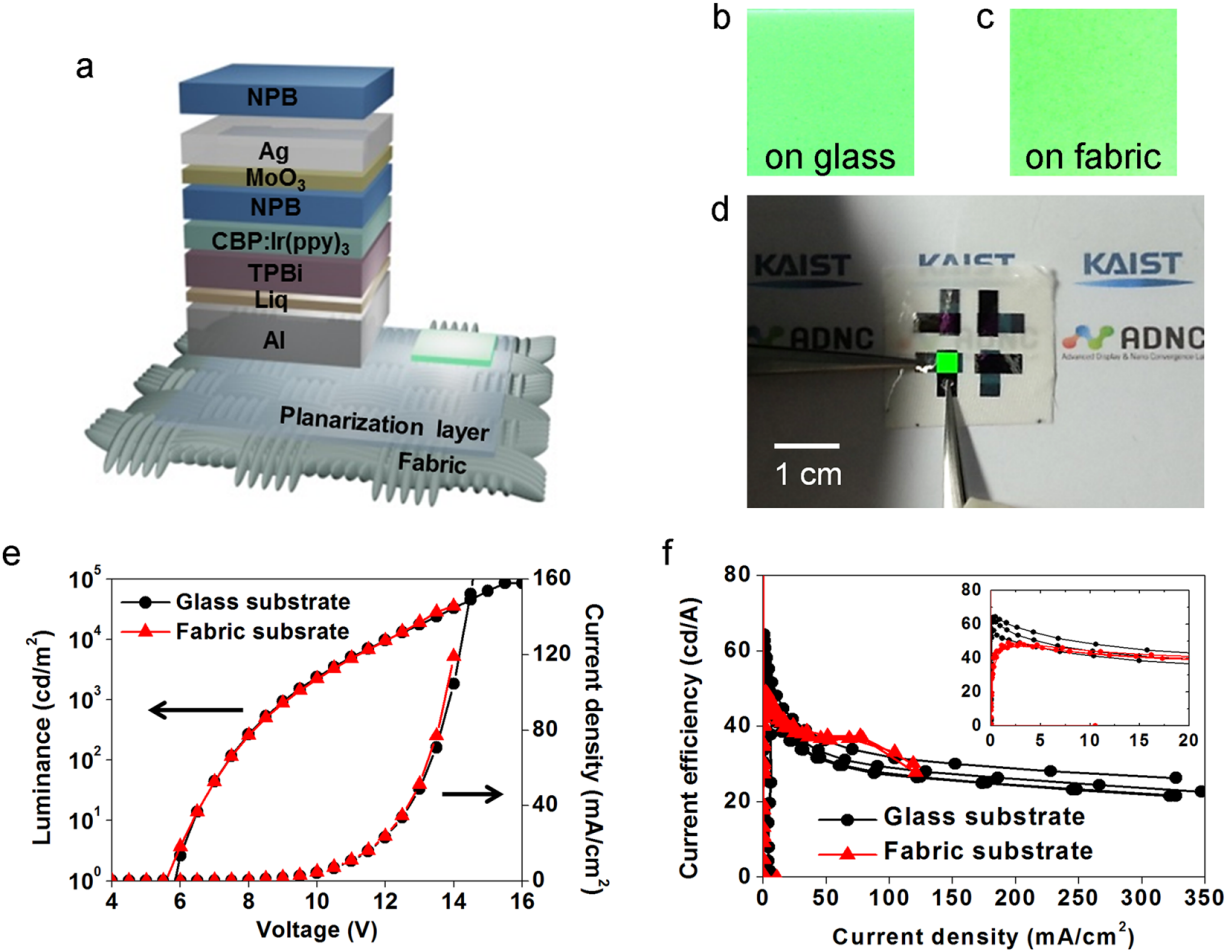
Electrical and optical characteristics of the fabric-based OLEDs: (**a**) Schematic illustration of the fabric-based OLED. (**b,c**) Images of emitting cells (**b**) on glass, (**c**) on the fabric. (**d**) Photograph of the emitting fabric-based OLED. (**e**) J-V-L characteristics of the glass- and fabric-based OLEDs. (**f**) Current efficiency levels of the glass- and fabric-based OLEDs (inset: enlarged graph in the small current density region).

**Figure 4:**
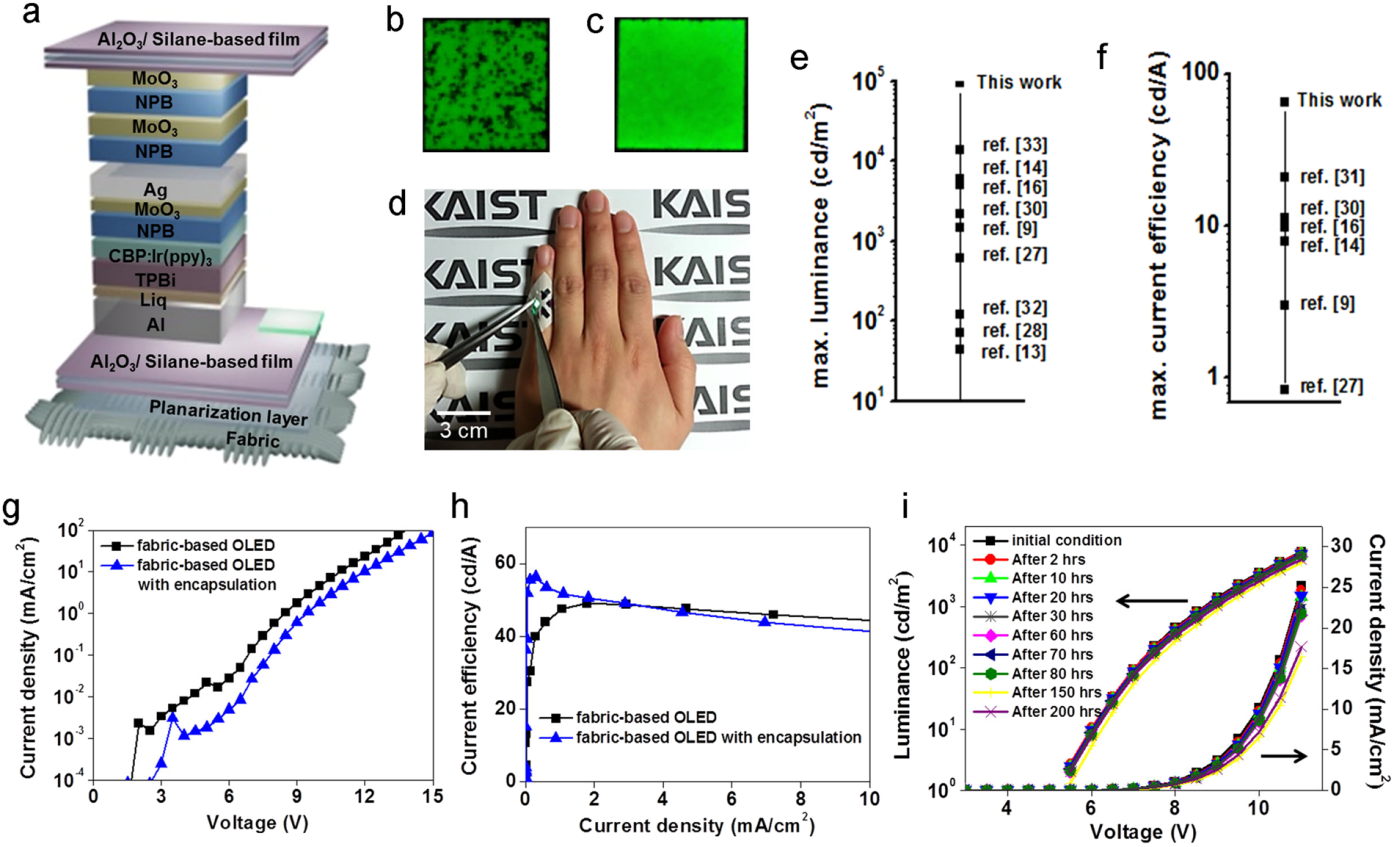
Optimized fabric-based OLED: (**a**) Schematic illustration of the optimized fabric-based, encapsulated OLEDs. (**b,c**) Images of emitting cells of the fabric-based, encapsulated OLEDs (**b**) without protective layers, (**c**) with protective layers. (**d**) Bending image of the optimized fabric-based device. (**e,f**) Ranking of wearable devices in terms of (**e**) the luminance and (**f**) the current efficiency. (**g**) J-V-L characteristics of the fabric-based OLEDs with and without encapsulation. (**h**) Current efficiency of the fabric-based OLEDs with and without encapsulation. (**i**) Deviations of the electrical-optical performance over time. The fabricated devices were placed in a constant temperature and humidity chamber (30 °C, R.H. 90%).

Figure 2a schematically illustrates the fabric-based OLED with top-emitting, micro-cavity structures^18, 19^ and a phosphorescent emission layer (EML)^20^. The fabric-based OLED consisted of two metal electrodes^21^, carrier injection and transport layers, an emission layer with a host-guest system, and an out-coupling layer. Cell images of the OLED on the fabric as well as glass are correspondingly showed in Fig. 2b and c. Both OLEDs emitted green light clearly without any defects. A photograph of the emitting OLED on the fabric is shown in Fig. 2d. The OLEDs on the fabric and the glass showed nearly identical electrical and optical performance levels (Fig. 2e and f). The maximum luminance and the current efficiency of the glass-based OLEDs were 169959 cd/m^2^ and 64.45 cd/A, while those of the fabric-based OLEDs were 93,030 cd/m^2^ and 49.14 cd/A, respectively. The difference between the current efficiency curves was caused by leakage current due to a number of abrupt peaks on the fabric surface. The planarized fabric showed a low average surface roughness value but a high peak-to-valley surface roughness (R_peak-to-valley_) value of 58.924 nm. This led to relatively low current efficiency of the fabric-based OLEDs in the small current region and fast degradation in the large current region. Nevertheless, the electrical and optical performance capabilities of the fabric-based OLEDs were sufficient for it to be used for indoor and outdoor displays, and the luminance and the current efficiency of the fabric-based OLEDs in this work were clearly high compared to those of previously reported wearable and fabric-based emitting devices (Fig. 4e and f). Changes in the EL peaks and color coordinates in relation to the emission angles are shown in Supplementary Figure S3. The angular dependency can be explained by the micro-cavity effects for higher out-coupling efficiency.

**Figure 3:**
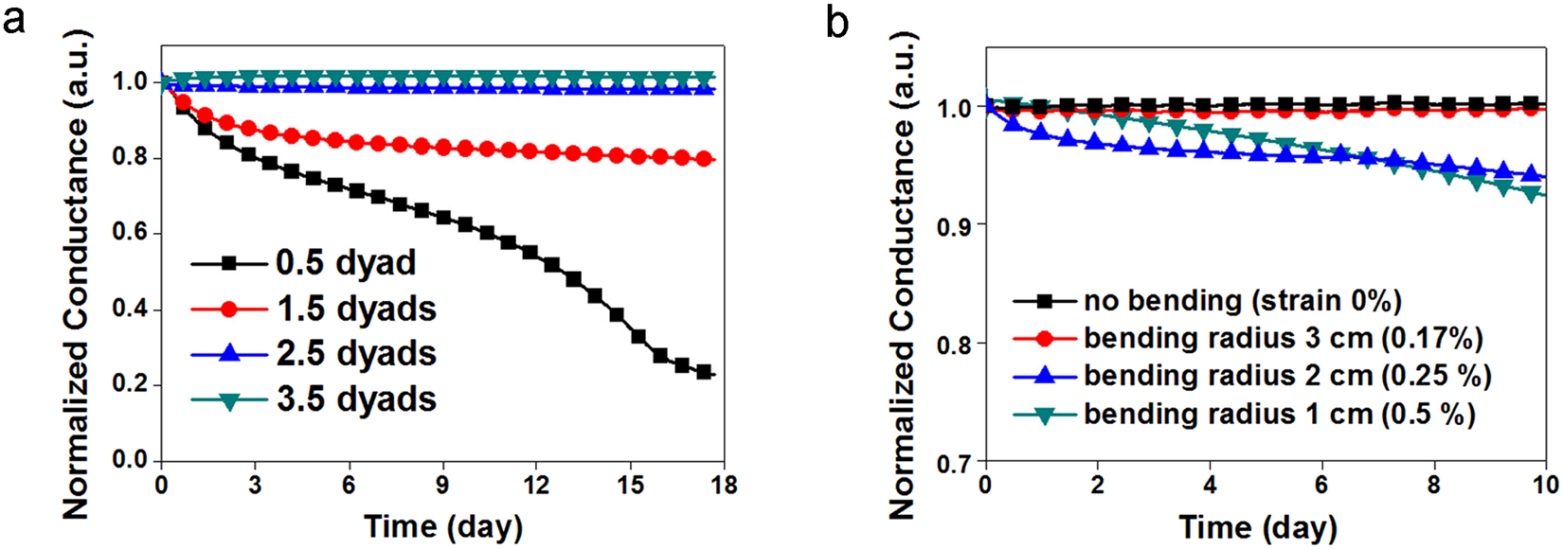
Calcium corrosion test: (**a,b**) Normalized conductance levels of a calcium pad over time, (**a**) according to the number of dyads, (**b**) according to the bending strain.

It is widely known that OLEDs are extremely sensitive to water vapor and oxygen. To operate OLEDs for extended periods of time, a moisture and oxygen barrier is vital^22–24^. The encapsulation for fabric-based OLEDs has to be flexible in order to work normally under mechanical stress conditions, and it must also be transparent to allow the light of the OLEDs to flow to the outside. A multi-barrier encapsulation composed of alternative moisture barriers and spacers is a prime solution^25^. The multi-barrier encapsulation in this work was fabricated with dense Al_2_O_3_ layers and soft silane-based polymer layers manufactured by Intech. The Al_2_O_3_ was formed using an atomic layer deposition (ALD) system^26^, and the silane-based solution was spin-coated. The optical transmittance levels of each layer and the fabricated multi-barriers exceeded 90% in the visible light region (Supplementary Figure S4). The barrier performance of the fabricated encapsulation was evaluated in terms of the water vapor transmission rate (WVTR, calculated according to the result of an electrical calcium corrosion test). As the number of dyads was increased, the absolute values of the slopes in a conductance-versus-time graph decreased, as shown in Fig. 3a. These results show that the Al_2_O_3_ layers obstructed the permeation of water vapor effectively. The data in Fig. 3a were converted into the WVTR values shown in Table 1, and the WVTRs of 2.5 and 3.5 dyads reached nearly 10^−6^ g/m^2^/day, nearly identical to the WVTR of commercialized glass lid encapsulation materials. The mechanical flexibility of the multi-barrier component was also investigated by cyclic bending tests (Fig. 3b and Table 2). The strain levels shown in Fig. 3b were calculated according to ε = d/2r (ε, strain; d, thickness, and r, bending radius), and 100-μm-thick PET was used as the base substrate to simplify the calculation of the strain. The multi-barrier structure even after 1000 cyclic bends with a radius of 2 cm (ε = 0.25%) showed an outstanding WVTR value of 8.98 × 10^−5^ g/m^2^/day.

**Table 1:** Results of the calcium corrosion tests: water vaper transmission rates according to the number of dyads.

The number of dyads	WVTR (g/m^2^/day)
0.5 dyad	1.15 × 10^−3^
1.5 dyads	1.40 × 10^−4^
2.5 dyads	8.87 × 10^−6^
3.5 dyads	4.96 × 10^−6^

**Table 2:** Results of the calcium corrosion tests after cyclic bending: water vapor transmission rates according to the bending radius and strain.

Bending radius (cm)	Strain (%)	WVTR (g/m^2^/day)
No bending	0	4.96 × 10^−6^
3 cm	0.17	8.87 × 10^−6^
2 cm	0.25	1.40 × 10^−4^
1 cm	0.5	1.15 × 10^−3^

The optimized structure of the fabric-based, encapsulated OLED for an emitting cloth was designed as shown in Fig. 4a. There are two important points with regard to the optimized device. First, MoO_3_/NPB/MoO_3_ layers were additively deposited to protect the OLED from water vapor and oxygen during the encapsulation process. After the thermal evaporation of the organic and metal layers, the OLED was completely exposed to air during the ALD and spin-coating processes; thus, the OLED was degraded. To prevent the degradation of the OLED, 150-nm-thick protective layers of MoO_3_/NPB/MoO_3_ were inserted. These protective layers delayed the permeation of H_2_O and O_2_ effectively, and cell images with or without the protective layers are shown in Fig. 4b and c. The second point is that the bottom side multi-barrier structure functioned as an additive planarization layer. As shown in Fig. 4g and h and in Supplementary Figure S5, the silane-based film effectively removed abrupt peaks on the fabric surface and reduced the peak-to-valley surface roughness. In fact, the fabric-based OLED with the multi-barrier encapsulation showed lower leakage current compared to that without the encapsulation. Due to the reduced electrical leakage, the fabric-based encapsulated OLED had higher current efficiency of 70.43 cd/A in comparison with the fabric-based OLED without encapsulation, as shown in Fig. 2. A photograph of the emitting fabric-based encapsulated OLED is shown in Fig. 4d. Compared to other wearable devices, the fabric-based OLEDs in this work showed outstanding performance, as shown in Figures 4e and 4f ^27–33^.

To measure the luminance variations over time, the fabricated device was placed in a constant-humidity constant-temperature chamber at 30 °C and R.H. 90%. As indicated in Fig. 4i and in Supplementary Figure S6, the luminance and the current efficiency over time tended to decrease gradually. This may have been caused by particle problems in the laboratory. As depicted in the cell images in Supplementary Figure S6, the initial defect grew over time. During the encapsulation process, the samples were exposed to numerous particles in the air and thus became contaminated. As a result, the current density decreased as the size of the non-emissive dark spot increased. Except for these defect spots, the OLED emitted green light stably.

**Figure 5:**
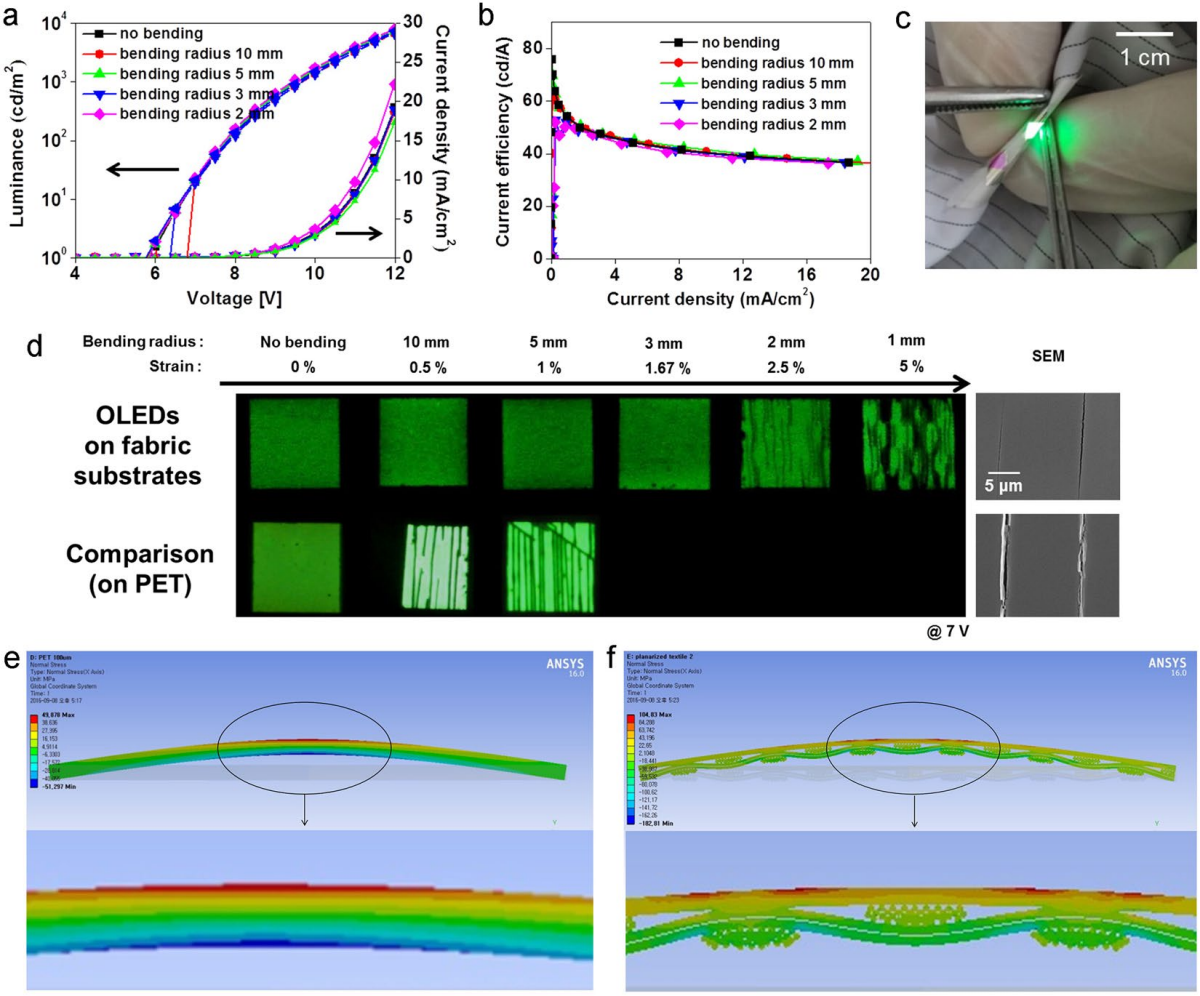
Bending strain: (**a,b**) Deviations of the electrical-optical performance capabilities according to the bending strain, (**a**) J-V-L characteristics, and (**b**) current efficiency. (**c**) Photograph of the emitting device under the bent condition. (**d**) Cell and SEM images of devices on the fabric and PET after the bending test. (**e,f**) Bending motion simulations using the finite element method (FEM), (**e**) two-dimensional film structure and enlarged image, and (**f**) two-dimensional planarized fabric structure and enlarged image.

The mechanical flexibility of the fabric-based OLEDs is one of the most important factors when attempting to realize wearable displays. Therefore, cyclic bending tests were done to apply mechanical stress to the fabric-based, encapsulated OLEDs. PET-based OLEDs were also used for comparison under identical conditions. The bending radii were infinite (no bending), 10, 5, 3, 2, and 1 mm (called folding), and the cyclic bending was repeated 1000 times. If the substrates are assumed to be 100-μm-thick films, the corresponding strains of the bending radii are 0, 0.5, 1, 1.67, 2.5 and 5%. The variations in the electrical and optical performance after cyclic bending are shown in Fig. 5a,b and d and in Supplementary Figure S7. After cyclic bending with bending radii of 10, 5 and 3 mm, the fabric-based OLEDs worked well without any abnormal operation in terms of the J-V-L characteristics and the current efficiency. For the bending radius of 2 mm, strong tensile stress caused faint cracks, vertically positioned in the bending direction, and the current efficiency decreased slightly. For the bending radius of 1 mm, only a cell image was taken, as the electrical and optical characteristics were not measurable owing to an electrical short caused by fatal cracks in the device. In Fig. 5d, SEM images after the bending of the sample with a bending radius of 1 mm for 1000 cycles showed that the cracks on the PET-based OLED cell were clear and thick. The widths of the cracks on the PET-based OLED were in the range of 700–900 nm, while cracks on the fabric-based OLEDs were thin and the crevices which formed on the fabric were no wider than 100 nm. The thicknesses of the fabric and the PET in this work were identical at 100 μm, but the mechanical stress and strain of the fabric were not determined only by the thickness. While the PET-based OLEDs with a film structure were vulnerable to bending strain, the fabric-based OLEDs having empty space within the fabric, movable fibers, and a wavy structure showed better strength characteristics, such as higher flexibility and lower stress. It is important to note that the crack patterns were similar to the weave patterns of the fabric, although the planarization sheet separated the fabric and the OLED. This phenomenon of the fabric-based OLEDs was analyzed by a structural simulation, as shown in Fig. 5e and f. In the film structure, when tensile and compressive stresses were applied to the convex and concave parts, a zero-stress region, referred to as a neutral axis, arose in the middle of the film. In contrast, the stress distribution within the fabric was complex. The empty space as well as each individual fiber and the woven structure influenced the stress distribution of the planarized fabric. The weave patterns of the fabric appeared on the OLED cell, indicating that the woven structure of the fabric affected the stress distribution of the OLED. This was verified by a cross-sectional, two-dimensional model of the fabric. The regions of the planarization layer in the free space between the fibers were under higher strain levels because there were no supporting fibers. Accordingly, the higher strain parts of the fabric-based OLED were damaged first.

**Figure 6:**
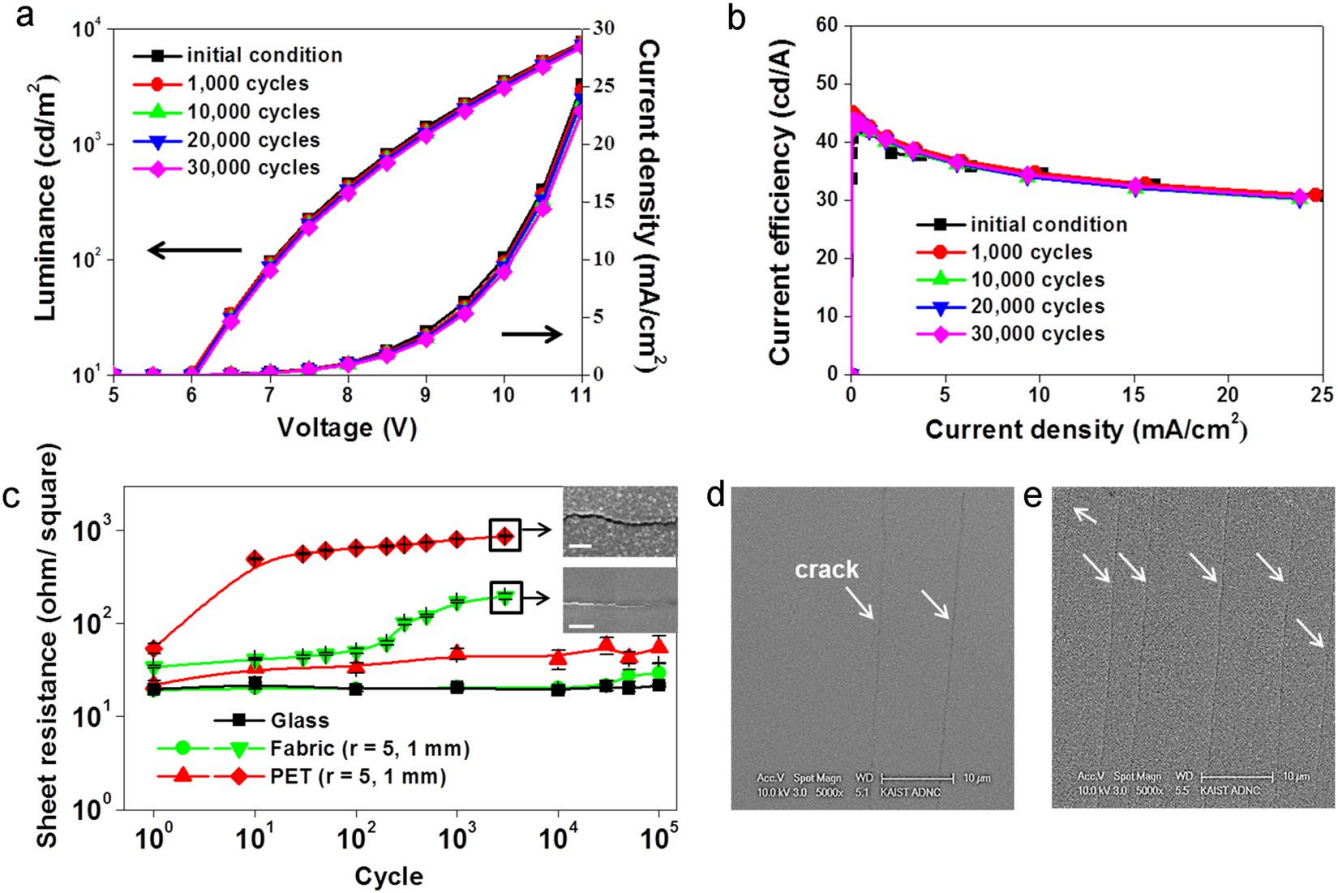
Bending fatigue: (**a,b**) Deviations of the electrical-optical performance capabilities according to the number of bends, (**a**) J-V-L characteristics, and (**b**) current efficiency. (**c**) Deviations in the sheet resistance of 100-nm-thick aluminum according to the bending strain and number of cycles (scale bars: 1 μm). (**d,e**) SEM image of Al on (**d**) the fabric, and (**e**) PET after bending with a bending radius of 1 mm at 3,000 cycles.

Figure 6 shows the results of an analysis of the bending fatigue of the fabric. As depicted in Fig. 6a and b, the fabric-based OLEDs retained their electrical-optical performance capabilities after bending with a bending radius of 1 cm for 30,000 cycles. In Fig. 6c, deviations of the sheet resistance of 100-nm-thick aluminum on the fabric and PET after cyclic bending were measured for a comparison of the bending fatigue of the fabric and PET. The initial sheet resistance levels of Al on the fabric and PET were 17.75 and 17.19 ohm/square before bending with a bending radius of 5 mm, respectively, and the corresponding values were 19.13 and 17.44 ohm/square before bending with a bending radius of 1 mm, respectively. The sheet resistance of Al on the fabric after extensive cycling with a bending radius of 5 mm for 100,000 cycles was 29.31 ohm/square, while that of the PET was 55.43 ohm/square. The sheet resistance of Al on the fabric increased by 172%, but that of PET rose by 324%. With a bending radius of 1mm, the corresponding sheet resistance levels of Al on the fabric and PET were 196 and 868 ohm/square after 3,000 cyclic bends. The sheet resistance levels of Al on the fabric and PET after bending increased by 10 and 51 times, respectively, compared to the initial conditions. The deviations of the sheet resistance due to Al oxidation were very minor. SEM images after extensive mechanical cycling at a bending radius of 1 mm and 3000 cycles are shown in Fig. 6d and e. The crack density of the Al on PET was three times higher than that on the fabric.

In summary, a highly efficient and flexible light-emitting fabric for clothing-shaped wearable displays was demonstrated. The fabric was woven from PEN fibers, and the planarization layer was thermally laminated onto the fabric. The planarized fabric showed an extremely low surface roughness of R_q_ = 2.073 nm. OLEDs were deposited by thermal evaporation, and transparent-flexible encapsulation with additive protective layers blocked the permeation of H_2_O and O_2_ effectively. The fabricated devices showed high maximum current efficiency of 70.43 cd/A, and luminance of 35,844 cd/m^2^. The device on the fabric operated stably after harsh bending, even at a bending radius of 2 mm for 3,000 cycles and a bending radius of 1 cm after 30,000 cycles. At a bending radius of 1 mm, leakage current occurred within the devices, and crack patterns, identical to the weave patterns of the fabric, appeared on the OLED cell. This phenomenon was analyzed in an ANSYS structural mechanics simulation. The proposed device is expected to be applied to various e-textile industries, such as in the manufacturing of curtains and tablecloths and in automobiles as well as in the fashion and healthcare industries in addition to serving as functional clothes. This work may contribute to the advancement of the wearable electronic industry as well as provide an improved understanding of and new horizons for various fabric-based devices.

## Methods

### Planarized fabric substrate

Soft fabrics were made by KOLON Glotech Inc. Heat-resistant polyethylene naphthalate (PEN) fibers were woven into the fabrics. The thickness of a fiber and the weaving periods between the woven fibers were approximately 11 μm and 520 μm, respectively. A thin planarization sheet was thermally laminated onto the fabric in order to form a flat surface compatible with OLEDs approximately 200 nm thick. Additionally, a gas barrier of 1.5 dyads was inserted into the sheet to prevent the permeation of water vapor and oxygen. The sheets showed a moisture barrier property of nearly 10^−3^ g/m^2^/day.

### OLED

OLEDs were designed as a top-emitting, micro-cavity structure to improve the external quantum efficiency with a host-guest energy transfer system to increase the internal quantum efficiency. A highly reflective Al cathode and a semi-transparent Ag anode generated strong micro-cavity effects, and the resonance wavelength was matched with the electroluminescent peak of an emitter^34^. The OLED consisted of a 100-nm-thick aluminium layer, a 1-nm-thick lithium quinolate (Liq) layer^35^, a 40-nm-thick 2,2′,2″-(1,3,5-benzinetriyl)-tris(1-phenyl-1-H-benzimidazole) (TPBi) layer, a 20-nm-thick 4, 4′-bis (carbazol-9-yl) biphenyl (CBP) layer doped with 8 wt% tris (2-phenylpyridine) iridium(III) (Ir(ppy)_3_), a 50-nm-thick N,N′-Bis (naphtanlen-1-yl)-N,N′-bis (phenyl)-benzidine (NPB) layer, a 5-nm-thick molybdenum trioxide (MoO_3_) layer^36^, and a 30-nm-thick silver layer. To improve the out-coupling of the OLED, an additional NPB layer was also deposited. All organics, inorganics, and metals were thermally evaporated in a vacuum chamber at a pressure of approximately 3 × 10^−6^ Torr.

The voltage and current were applied to the OLEDs by a Keithley 2400 sourcemeter, and the luminance and the color coordinates were measured by a CS2000 device by Konica Minolta. Cell images of the OLEDs were taken by a GE-5 digital microscope by View Solution Inc.

### OLED encapsulation

The multi-barriers for OLED encapsulation were fabricated by the alternative deposition of inorganic and organic layers. The inorganic layers, Al_2_O_3_, were formed by an atomic layer deposition (ALD) system using trimethyl aluminium (TMA) as a precursor and H_2_O as a reactant. TMA and H_2_O gases were injected for 0.1 second each, and the N_2_ purges used between source injections were 10 seconds. This process was repeated for 330 cycles to deposit a 30-nm-thick Al_2_O_3_ layer. The process temperature and pressure in the ALD chamber were 70 °C and 2 × 10^−3^ Torr. The organic, a silane-based solution, was spin-coated under acceleration for 30 seconds at 5,000 rpm for 3 seconds and then dried in an ALD chamber for 10 minutes.

The water vapor transmission rate (WVTR) values of the OLED encapsulations were evaluated from the results of electrical calcium corrosion tests^37^. The calcium pads encapsulated by the multi-barrier structure were placed in a humidity chamber at a constant temperature of 30 °C and 90% R.H. and the decrease in the electrical conductivity of the calcium pad was then measured.

### Device Characterization

Cyclic bending of the OLEDs was repeated by a bending machine. The ANSYS structural mechanics software using the finite-element method (FEM) was used to analyze the mechanical stress distributions of a fabric and a film modelled in 2 dimensions. The Young’s modulus and Poisson’s ratio of the models were 3.5 GPa and 0.3, respectively, which were the measured values of conventional polyethylene terephthalate (PET) from a nano-indentation system (Nano Indenter XP, U.S.A.).

## Electronic supplementary material

Supplementary Information

Supplementary movie
