# Supplementary material for: Highly Flexible and Efficient Fabric-Based Organic Light-Emitting Devices for Clothing-Shaped Wearable Displays
Source: Sci Rep. 2017 Jul 25;7:6424. doi: 10.1038/s41598-017-06733-8 (PMC5526867; doi:10.1038/s41598-017-06733-8)
Supplement: Supplementary file 1 — Supplementary Information [file 41598_2017_6733_MOESM1_ESM.pdf]

Supplementary Information

Highly Flexible and Efficient Fabric-Based Organic Light-Emitting Devices for Clothing-Shaped Wearable Displays

Seungyeop Choi, Seonil Kwon, Hyuncheol Kim, Woohyun Kim, Jung Hyun Kwon, Myung Sub Lim, Ho Seung Lee and Kyung Cheol Choi*

Supplementary Figure S1

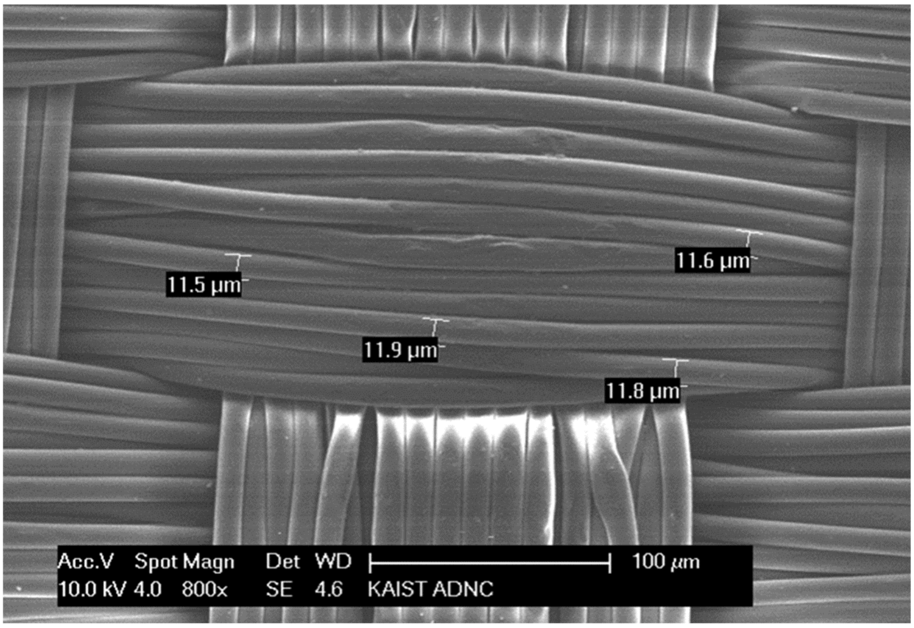

Figure S1. SEM image of the fabric used in this work.

Supplementary Figure S2

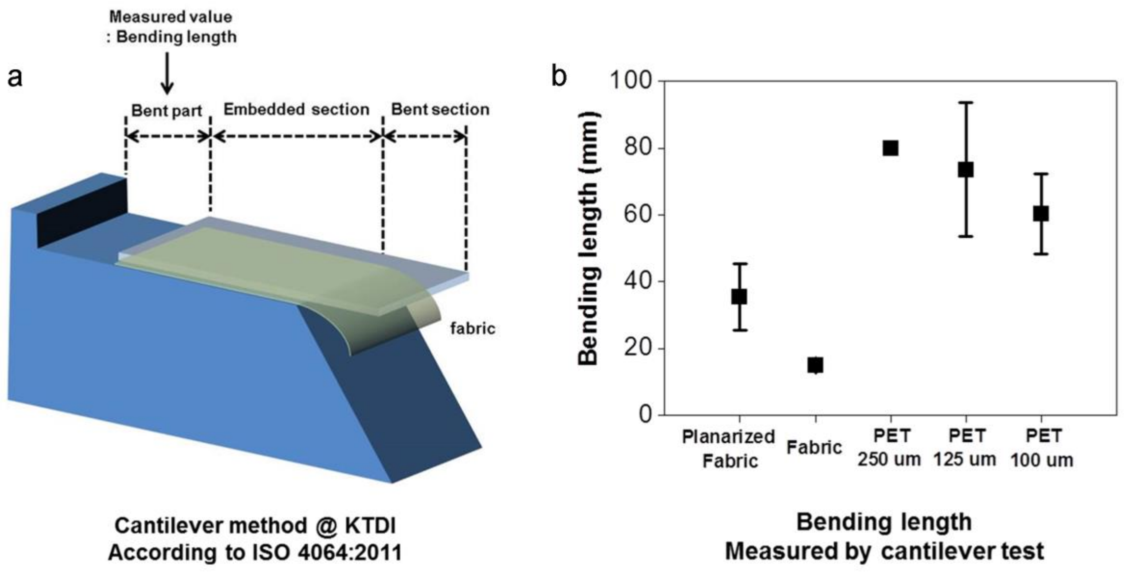

Figure S2. Cantilever test. (a) Schematic diagram of the cantilever test. (b) Bending lengths according to the kinds of substrates. The angle of the slope in the cantilever tester is 41.5°. As the bending length becomes smaller, the substrate becomes more flexible. The planarization layer affected the flexibility of the fabric; however, the planarized fabric is still more pliable than even the thinner plastic substrate.

Supplementary Figure S3

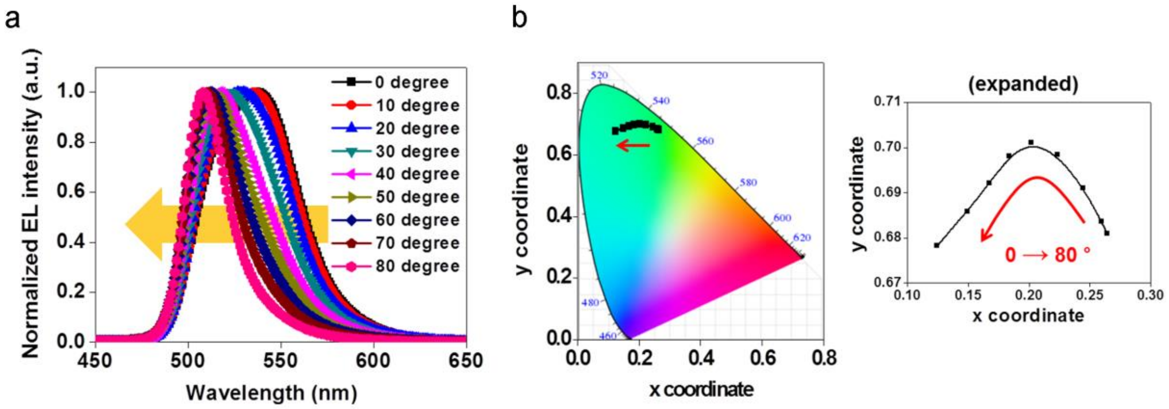

Figure S3. Changes in the EL peaks and color coordninates in relation to the emission angles of the fabric-based OLEDs

Supplementary Figure S4

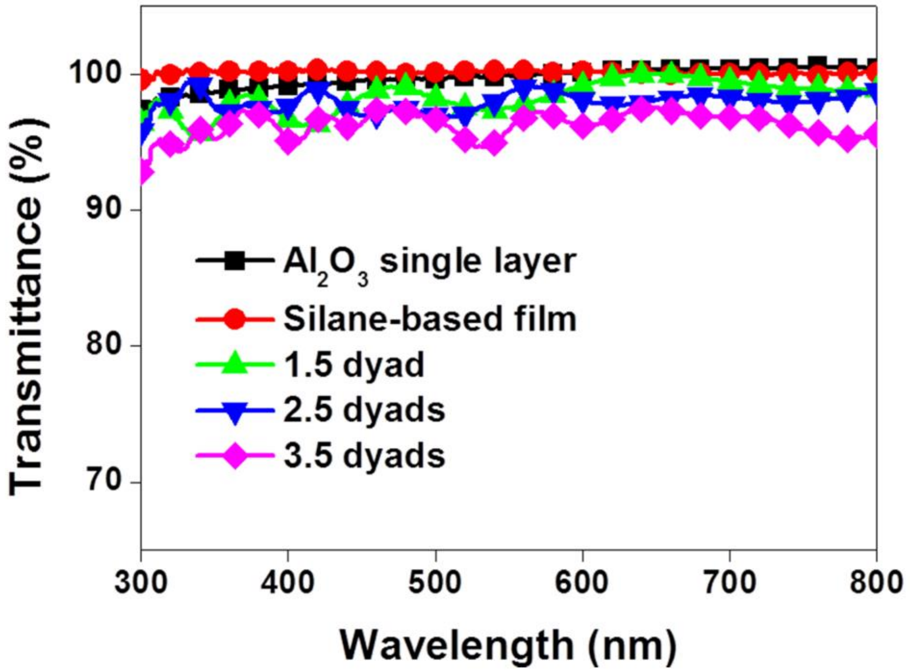

Figure S4. Optical transmittance of each layer included in the multi-barrier encapsulation.

Supplementary Figure S5

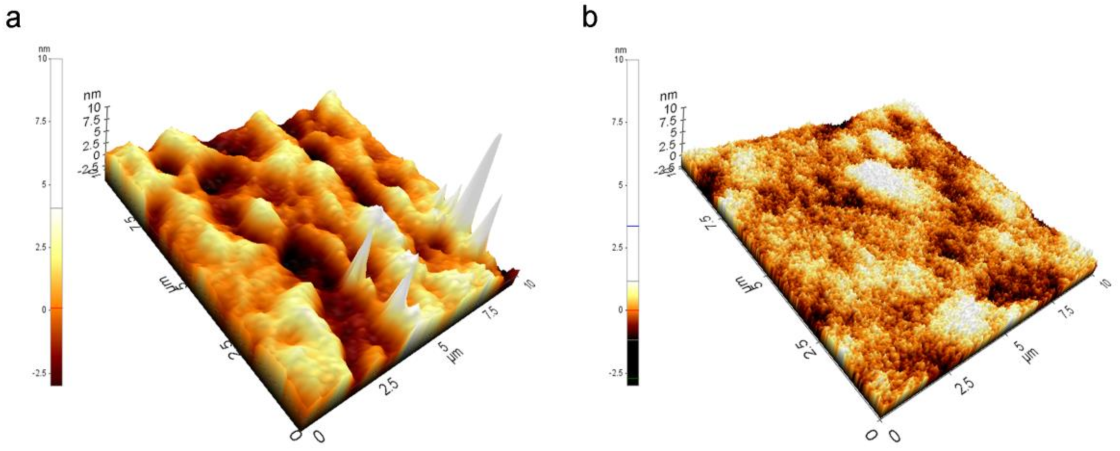

Figure S5. The relationship between the surface morphologies of the fabrics. (a) AFM result of the planarized fabric. (b) AFM result of the planarized fabric after an additive spin-coating of the silane-based film. Note that the multi-barrier of the bottom side affected the surface morphologies of the fabric substrates.

Spin-coating of silane-based film	Before	After
R_q (nm)	2.073	0.602
R_a (nm)	1.511	0.443
$R_{peak\text{-}to\text{-}valley}$ (nm)	58.924	6.068

Table S1. Specifications of the AFM results (Figure S5a and S5b). Root mean square (R_q), average (R_a), and peak-to-valley ($R_{peak\text{-}to\text{-}valley}$) surface roughness values of the fabrics before and after the spin-coating of the silane-based film.

Supplementary Figure S6

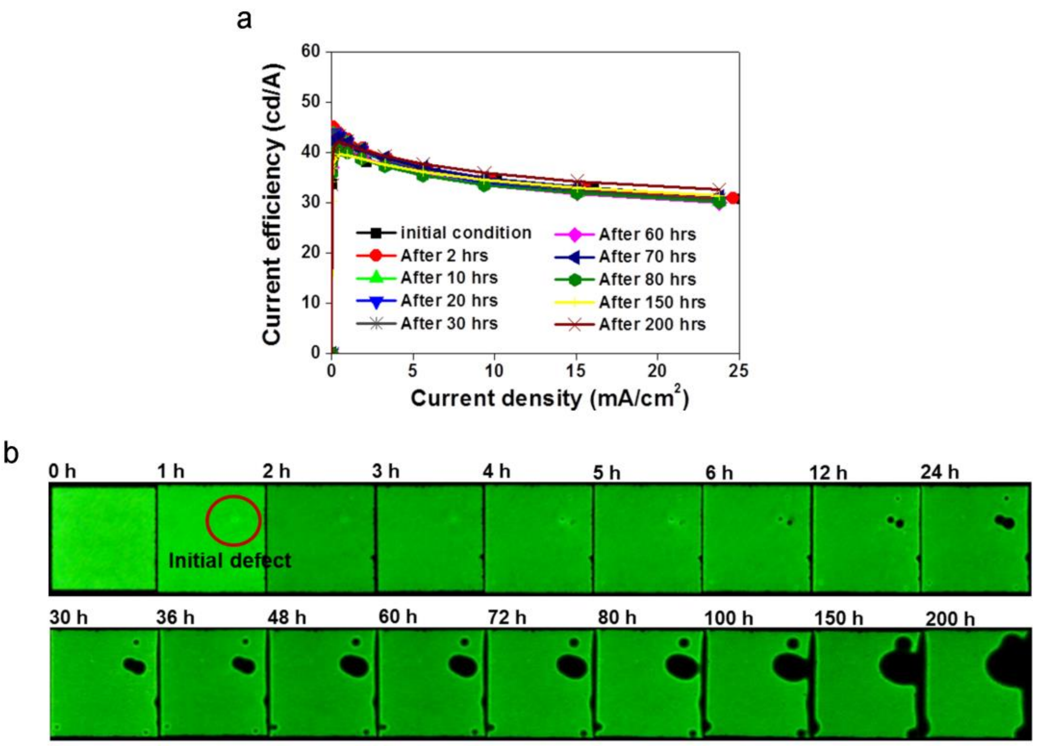

Figure S6. Current efficiency variations over time. Fabricated device was placed in a constant-humidity constant-temperature chamber at 30 °C and R.H. 90%.

Supplementary Figure S7

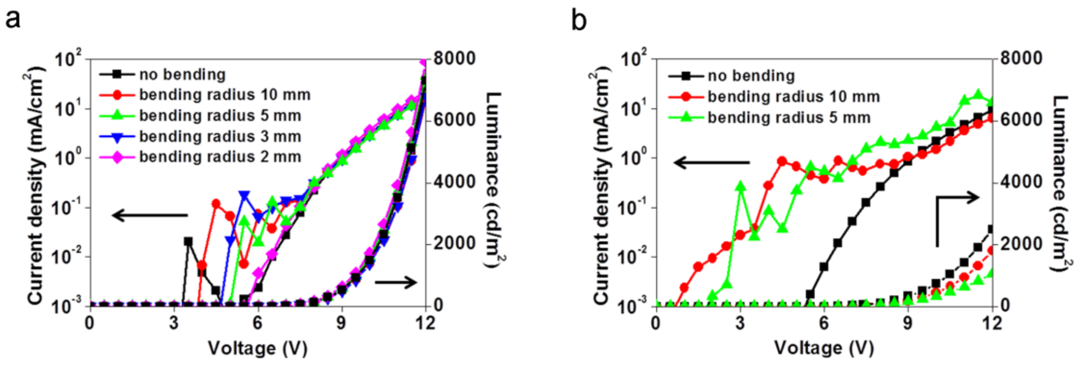

Figure S7. Veriations in electrical and optical performance after cyclic bending of the fabric-based and PET-based OLEDs.
